# Repeated dose toxicity and immunogenicity studies in SD rats: a new recombinant zoster vaccine (CHO cell) formulated with adjuvant BC02

**DOI:** 10.3389/fcimb.2026.1756776

**Published:** 2026-05-22

**Authors:** Qian Yang, Luxi Yang, Aihua Zhao, Lingyun Ge, Fang Liu, Zhengbiao Yang, Yue Huang, Qiuhong Jiang, Yaru Deng, Xuehan Hu, Jiahong Wang, Lijuan Xia, Suqin Zhang, Faqin Liang, Wenxin Lu, Kejun Zhu, Pansheng Xu, Xiaodong Tan, Wenhai Huang

**Affiliations:** 1 Center of Safety Evaluation and Research, Hangzhou Medical College, Hangzhou, China; 2 Zhejiang Provincial Key Laboratory of Drug Discovery and Safety Evaluation for Inflammatory Chronic Diseases, Hangzhou Medical College, Hangzhou, China; 3 Division of Tuberculosis Vaccine and Allergen Products, Institute of Biological Product Control, National Institutes for Food and Drug Control, Beijing, China; 4 Key Laboratory for Quality Research and Evaluation of Biological Products, National Medical Products Administration (NMPA), Beijing, China; 5 Key Laboratory of Research on Quality and Standardization of Biotech Products, National Health Commission (NHC), Beijing, China; 6 Anhui Zhifei Longcom Biopharmaceutical Co., Ltd., Hefei, China

**Keywords:** GLP, herpes zoster, immune response, recombinant zoster vaccine, repeated-dose toxicity study

## Abstract

Herpes zoster, an infectious disease resulting from the reactivation of the varicella-zoster virus, is characterized by intense neuropathic pain. Safe and effective vaccination remains the optimal strategy for preventing and reducing associated complications. Recombinant zoster vaccine (CHO cell) (Qiuhong et al) employs a novel adjuvant system, BC02. This study aimed to conduct a comprehensive non-clinical safety assessment of RZV in adult Sprague-Dawley rats to support its entry into clinical trials. The rats received four (sp, one additional dose beyond the intended clinical three-dose schedule) doses of RZV. Safety was assessed using general health observations, immunotoxicity and immunogenicity assays, hematological and biochemical profiling, and histopathological evaluations. Aside from the changes observed in the adjuvant control group, no significant toxic effects were detected. No distinct target organs of toxicity were identified. The no-observed-adverse-effect level (NOAEL) was 1.0 mL per rat, which is equivalent to two human doses. The vaccine also elicited strong cellular and humoral responses, indicating its favorable immunogenicity. In summary, this systematic toxicology program supports the safety, tolerability, and immunogenicity of RZV and justifies its progression to human clinical trials.

## 1 Introduction

Herpes zoster (HZ), commonly known as shingles, is a painful cutaneous eruption caused by the reactivation of latent varicella-zoster virus (VZV) in sensory ganglia (Schmader, 2018). This reactivation typically occurs in individuals with declining VZV-specific cell-mediated immunity, often associated with aging or immunosuppression (Mbinta et al., 2022). A significant complication of HZ is postherpetic neuralgia (PHN). This persistent and often debilitating neuropathic pain can last for months or even years after the rash has resolved, substantially impairing the quality of life of affected individuals.

HZ has emerged as a significant global public health concern. According to a 2023 data analysis, the pooled incidence rate of HZ in China was 4.28 per 1,000 person-years, increasing with age to 11.69 per 1,000 person-years among individuals aged ≥ 60 years (Zhang et al., 2023). A major complication of HZ is postherpetic neuralgia (PHN). This persistent and often debilitating neuropathic pain can last for months or even years after the rash has resolved, substantially impairing the quality of life of affected individuals. Among patients with HZ, 29.8% develop PHN (Yang et al., 2019). Key risk factors for HZ onset include advanced age and immunocompromised conditions resulting from chronic metabolic diseases, immune system disorders, cancer, and other diseases. Although antiviral therapy can reduce the severity and duration of acute zoster, it may ineffectively lower the incidence of PHN or other complications (Chen et al., 2014). With the ongoing global trend of population aging, the incidence of HZ is expected to rise, posing an increasing economic burden on healthcare systems (Jin et al., 2025). Recently, HZ vaccines have become an essential tool for preventing HZ and its complications (Harbecke et al., 2021). Developing these vaccines employs a range of technologies, including classical live-attenuated and inactivated vaccines, as well as newer recombinant subunit methods.

The recombinant zoster vaccine induces immunity through recombinant VZV antigenic proteins, thereby eliminating the requirement for intact viral particles. Current recombinant zoster vaccines primarily target glycoprotein E (gE), the most abundant VZV envelope protein (Cole and Grose, 2003; Yao et al., 1993). Functionally, gE forms a heterodimer complex with gI, acting as an Fc receptor in infected cells, facilitating viral recognition, dissemination, and immune evasion (Yao and Grose, 1994). The close association of gE with host infection mechanisms renders it an optimal antigenic target for HZ vaccine design (Cunningham et al., 2023). Unlike live attenuated vaccines, recombinant zoster vaccines eliminate the potential for vaccine-strain breakthrough infections or reactivation while effectively eliciting targeted antibody production and cell-mediated immunity (CMI) responses. Many national health systems now designate the recombinant zoster vaccine as the primary or preferred approach for preventing herpes zoster (Mwakingwe-Omari et al., 2023).

Effective vaccination requires not only the core antigen but also adjuvants that can accurately enhance and modulate immune responses (Ben-Akiva et al., 2025; Zhao et al., 2023). Adjuvant systems that combine traditional adjuvants with immune stimulants leverage components with distinct mechanisms of action to produce synergistic effects. Through multi-mechanistic and multi-target approaches, these systems elicit stronger, more durable, and more balanced immune responses (Roman et al., 2024; Firdaus et al., 2022).

The “recombinant zoster vaccine (CHO cell)” developed by Anhui Zhifei Longcom Biopharmaceutical Co., Ltd. incorporates a novel adjuvant system designated BCG CpG DNA compound adjuvants system 02 (BC02) (Qiuhong et al., 2024), which is composed of Al(OH)3 inorganic salt adjuvant and BC01 (BCG CpG DNA compound adjuvants system 01), a Toll-like receptor 9 (TLR9) agonist (Xia et al., 2025). BC02 is characterized by an innovative manufacturing process and a ready-to-use formulation designed to work synergistically with the antigen. Its unique mechanism of action enables diverse immunoenhancing effects, including reduced antigen dose requirements, accelerated immune response onset, and prolonged immunity (Ishihara et al., 2024).

As a novel vaccine candidate, a comprehensive preclinical safety evaluation is an indispensable prerequisite for clinical translation. Such an assessment should not only investigate potential toxicities but also characterize the vaccine’s inherent pharmacological effects, such as localized inflammatory responses and systemic immune activation. These are the expected consequences of effective immunization. This study was designed to conduct a thorough non-clinical safety assessment of this vaccine. Following the International Council for Harmonization of Technical Requirements for Pharmaceuticals for Human Use (ICH) guidelines and the National Medical Products Administration (NMPA, Chinese) guidelines and mirroring the clinical three-dose schedule, we conducted a study in Sprague-Dawley (SD) rats and mirroring the clinical three-dose schedule, we conducted a study in Sprague-Dawley (SD) rats using four intramuscular injections (muscle groups of the hind limbs on both sides) of RZV (N + 1 design, one additional dose beyond the intended clinical three-dose schedule). We characterized adverse reactions based on their nature, severity, dose-response, time-response, and reversibility. We also assessed immunogenicity and immunotoxicity. This study aimed to identify potential target organs or tissues after repeated dosing and establish a no-observed-adverse-effect level (NOAEL). The results are intended to provide reference data from animal studies for clinical research and highlight key parameters that may require close monitoring during clinical trials.

## 2 Materials and methods

### 2.1 Study design

**Figure 1:**
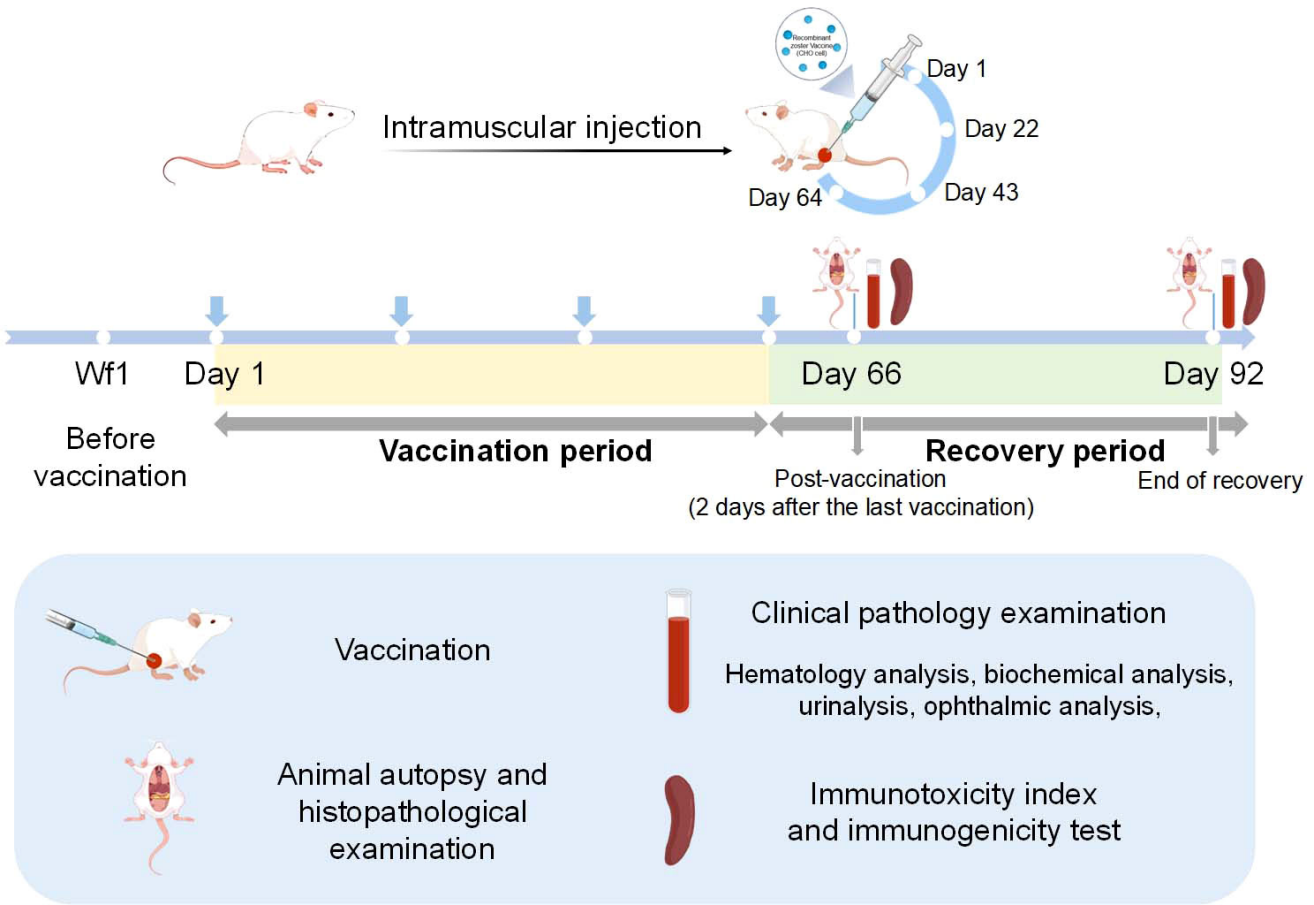
Overview of 4-dose repeated administration toxicity test, concomitant immunogenicity, and immunotoxicity of recombinant zoster vaccine (CHO cell) via intramuscular injection in rats.

The study design is illustrated in **Figure 1**. A total of 120 SD rats (60 males and 60 females) were randomly assigned to four experimental groups: a blank control group (0.9% sodium chloride solution, 1.0 mL/rat), an adjuvant control group (1.0 mL/rat), a low-dose group (0.5 mL (one human dose)/rat), and a high-dose group (1.0 mL (two human doses)/rat). This dose selection was based on the guidelines for non-clinical repeated-dose toxicity studies of vaccines issued by the ICH and the China National Medical Products Administration (NMPA).

The vaccine (VZV-gE, 59 μg/dose, per 0.5 mL, equivalent to 1 human dose; Batch No. 202212001) and adjuvant (BCG-CpG-DNA, 101 μg/dose, per 0.5 mL, equivalent to 1 human dose, without VZV-gE; Batch No. 202405001) were developed and provided by Anhui Zhifei Longcom Biopharmaceutical Co., Ltd. Specific pathogen-free (SPF) SD rats (male body weight: 256–329 g; female body weight: 234–266 g) were procured from Zhejiang Vital River Laboratory Animal Technology Co. Ltd. (SCXK (Zhe) 2024-0001; Quality Certification No. 20240613Aazz0619018706 (♂), No. 20240613Aazz0619018199 (♀)). This study was conducted at the Safety Evaluation Research Center of Hangzhou Medical College (SYXK (Zhe) 2022-0027), a facility accredited by the Association for Assessment and Accreditation of Laboratory Animal Care (AAALAC) International. Rats were housed by gender (≤ 5 animals per cage) with ad libitum access to standard rodent diet and locally sourced, softened, and filtered water. Ambient conditions were maintained throughout the study at 21.4–25.2 °C, 46.0%–70.6% relative humidity, and a 12-h light/dark cycle. All animal studies and procedures were conducted in accordance with the institutional guidelines for the care and use of laboratory animals. They were approved by the Institutional Animal Care and Use Committee (IACUC) (Approval No.: GLP-2024-096).

### 2.2 Clinical observation and routine measurement endpoints

Throughout the study, the rats were observed and recorded daily for external signs, including general appearance, condition of the vaccination site, behavioral activity, skin integrity, respiration, oral and nasal discharge, glandular secretion, fecal and urinary characteristics, and mortality. Body weight and food consumption were measured weekly. Ophthalmic examinations and urinalysis were performed on all surviving animals prior to dosing, at the Post-vaccination period (2 days after the last vaccination), and at the end of the recovery period (28 days after the last vaccination). At the Post-vaccination and end of recovery periods, rats were anesthetized and euthanized via intraperitoneal injection of 50 mg/kg Suthan^®^ 50 solution (concentration 50 mg/mL). Subsequently, the following assessments were conducted: hematology (including coagulation), serum biochemistry, immunotoxicity and immunogenicity evaluations, bone marrow cell morphology analysis, weights and organ-to-body weight ratios of major organs, gross necropsy, and histopathological examination. Hematological and serum biochemical parameters were analyzed using an XN-1000V Hematology Analyzer (Bayer, USA) and HITACHI 7180 Automatic Biochemistry Analyzer (HITACHI, Japan), respectively. Coagulation parameters, including activated partial thromboplastin time (APTT), fibrinogen (Fbg), and prothrombin time (PT), were measured using a Sysmex CA-1500 analyzer (Sysmex, Japan). Flow cytometry (FACS Canto II, USA) was used to investigate the immunophenotypes of peripheral blood lymphocytes, including CD3^+^, CD4^+^, and CD8^+^ T lymphocytes. Urine was analyzed using a urinalysis instrument (DIRUI N-600, China), and ophthalmic examination was performed using a Welch Allyn 12500 ophthalmoscope (USA).

### 2.3 Immunogenicity and immunotoxicology evaluation

Splenic samples collected at the post-vaccination period (day 2 after the final immunization) and at the end of the recovery period (day 28 after the final immunization) were subjected to cellular immunity assays. In contrast, serum samples were used for humoral immunity evaluation.

#### 2.3.1 IgG antibody GMT assay

VZV gE protein (1 μg/mL) was coated onto the 96-well plates. Serially diluted serum samples were added, followed by HRP-conjugated goat anti-rat IgG. TMB substrate was used for the color development. Optical density (OD_450_) was measured, and the geometric mean titer (GMT) was calculated.

#### 2.3.2 Fluorescent antibody to membrane antigen assay

The culture medium from each T75 cell culture flask was discarded. A total of 20 mL of maintenance medium and 100 μL of varicella-zoster virus were added, and the cultures were incubated at 5% CO_2_. When approximately 60% cytopathic effect was observed, the cells were used for the FAMA assay. The cells were resuspended at a density of 6 × 10^5^ cells/mL. Serum samples were serially diluted from 1:4 to 1:128. Fluorescein isothiocyanate conjugated immunoglobulin G (FITC-IgG) was added at a 1:1000 dilution. The preparations were examined using fluorescence microscopy. A complete green fluorescent ring around VZV-infected cells was interpreted as positive. The reciprocal of the highest serum dilution that formed a complete ring was recorded as the VZV-specific antibody titer. The absence of a complete ring was considered to be negative. If no ring was observed at the lowest dilution (1:4), the VZV-specific antibody titer was recorded as 2 for statistical analysis. The mean antibody titer for each group was expressed as the geometric mean titer (GMT), calculated using the same method as for the specific IgG antibody GMT. Seroconversion was defined as samples with a VZV-specific antibody titer ≥ 4. The seroconversion rate was defined as the proportion of animals with a VZV-specific antibody titer ≥ 4 detected by FAMA relative to the total number of animals in that group.

#### 2.3.3 VZV gE antigen-specific rat interferon gamma, interleukin-2, and tumor necrosis factor-alpha cytokine spot assay

A gE-specific cytokine enzyme-linked immunospot (ELISPOT) assay was conducted. Splenocytes were isolated and added to pre-coated ELISPOT plates, followed by stimulation with gE polypeptide pools. The gE peptide pool utilized in the ELISPOT assay was derived from the full-length gE protein sequence (623 amino acids). This peptide library comprises 134 peptides, each 15 amino acids in length, with an overlapping region of 11 amino acids between adjacent peptides. The working concentration of the peptide pool was 0.625 μg/mL. The number of spots representing gE antigen-specific rat IFN-γ, IL-2, and TNF-α-secreting cells was quantified. The antigen-specific spot count was calculated by subtracting the number of spots in the negative control wells from the total number of spots observed after peptide stimulation. Data from the different groups were aggregated. Statistical methods were used to analyze the differences between the groups.

Immunotoxicity evaluation was conducted based on three aspects: immune organs, immune cells, and immune-active molecules. The spleen and thymus were examined for weight, organ-to-body weight ratios, and histopathological changes. Furthermore, histopathological examination was performed on relevant lymph nodes and bone marrow.

Serum C3 and IgG concentrations were quantified by immunoturbidimetry using a HITACHI 7180 automated analyzer (Hitachi, Tokyo, Japan). Peripheral blood lymphocytes were assessed, including the measurement of CD3^+^, CD4^+^, and CD8^+^ T-cell subsets and the CD4^+^/CD8^+^ ratio.

### 2.4 Necropsy and histopathology

On the day following the fourth vaccination, 20 SD rats from each group (n = 10/gender) were euthanized, while the remaining animals were sacrificed 28 days after the final vaccination (n = 5/gender). A gross necropsy was performed immediately after euthanasia. External features, skin, natural orifices, tissues, and organs were examined, and gross findings were recorded. The weights and organ-to-body weight ratios of major organs, including the brain, visceral organs, glands, bones, lymphoid tissues, reproductive organs, and vaccination sites, were recorded for each animal. Eyeballs, Harderian glands, testes, and epididymides were preserved in Davidson’s fixative, whereas all other tissues were stored in 10% neutral buffered formalin. All tissue samples were embedded in paraffin, stained with hematoxylin and eosin (H&E), and examined microscopically.

### 2.5 Statistical analysis

Statistical analyses were performed using the Statistical Package for the Social Sciences software (version 18.0). Continuous variables, including body weight, growth rate, organ weights, organ-to-body weight ratios, hematological and biochemical parameters, and immunological indices, are summarized as mean ± standard deviation. Between-group differences were tested using one-way ANOVA. When Levene’s test indicated homogeneous variances, least significant difference (LSD) post-hoc tests were applied; when variances were unequal, Games–Howell tests were used to analyze the data. The significance threshold was set at P < 0.05.

Qualitative data, such as urine test indices, were reported as frequency scores (number of occurrences in total samples). Non-parametric differences were evaluated using the Kruskal–Wallis test. When overall significance was detected at P < 0.05, post-hoc pairwise comparisons were conducted.

## 3 Results

### 3.1 Clinical observations

Throughout the study, no remarkable abnormalities or mortality were observed in any of the rats in the blank control and low-dose groups. In adjuvant control and high-dose groups, swelling was palpable at the vaccination site in male and female rats following each dose, which subsequently subsided gradually. The swelling was no longer palpable on days 13, 29, 49, and 73 after doses were administered on days 1, 22, 43, and 64, respectively. No other abnormalities or mortality were observed in these groups during the remainder of the study.

**Figure 2:**
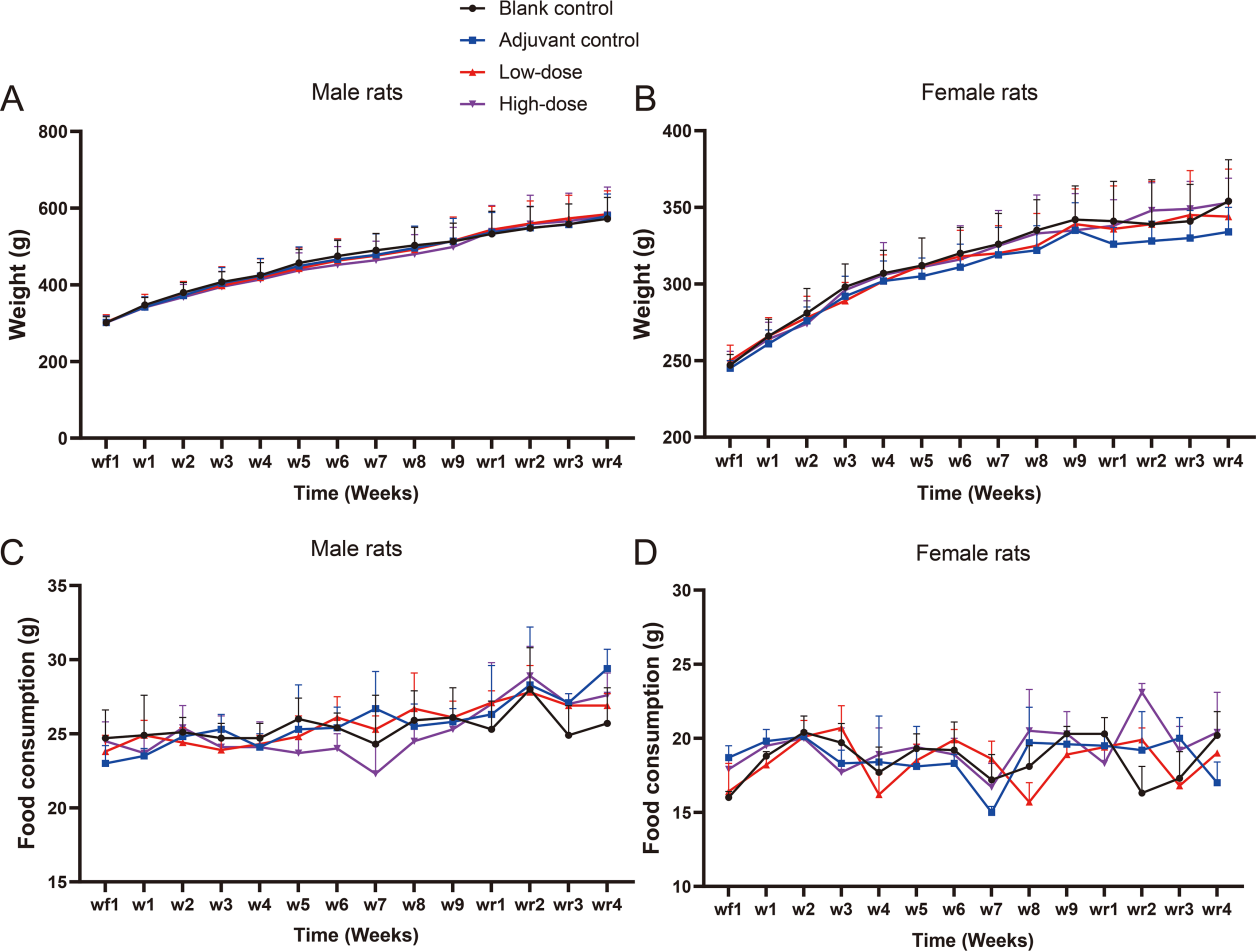
Clinical observation of rats during the toxicity test of repeated administration of four doses of recombinant zoster vaccine (CHO cell) intramuscularly. **(A, B)** Body weight and **(C, D)** food consumption of rats in the long-term toxicity study. Post-vaccination period (n = 15/gender) and end of the recovery period (n = 5/gender).

Compared with the blank control group, body weight gain in all dose groups was unaffected throughout the experimental period, with male and female rats demonstrating progressive increases in body weight over time (**Figures 2A, B**). No significant differences in food consumption were observed among the groups during the study (**Figures 2C, D**). Collectively, these findings indicate that RZV was well-tolerated and had no adverse effects on general health, growth, or feeding behavior.

### 3.2 Hematological and serum biochemical analysis

Compared with the blank control group, at the post-vaccination period, increases in neutrophils (NEUT) were observed in female and male rats of the vaccine high-dose group, as well as in female rats of the adjuvant control and low-dose groups. Moreover, slight elevations in white blood cell count (WBC), monocytes (MONO), eosinophils (EOS), and basophils (BASO) were observed in female rats of the low-dose group. After the 4-week recovery period, all these changes returned to baseline, except for elevated eosinophil counts (#EOS) in female rats in the low- and high-dose vaccine groups (**Tables 1**, **2**). The mild alterations in leukocyte-related parameters described above were considered related to the inflammatory and immune responses triggered by vaccine vaccination. At the post-vaccination period, a rising trend in fibrinogen (Fbg) was observed in female and male rats of the adjuvant control, vaccine low-dose, and high-dose groups, displaying a certain dose-response relationship. This change was no longer present by the end of the recovery period. It was interpreted as being associated with the enhanced immune response following vaccination rather than representing a toxicological effect.

**Table 1:** Hematological analysis of female SD rats treated with herpes zoster vaccine (CHO cell).

Parameter	Post-vaccination period (n = 10)												End of the recovery period (n = 5)											
	Blank control			Adjuvant control			Low-dose			High-dose			Blank control			Adjuvant control			Low-dose			High-dose		
WBC (10^9^/L)	5.12	±	1.03	5.99	±	1.12	6.87	±	1.59**↑	6.25	±	1.34	5.73	±	2.65	3.81	±	0.61	5.83	±	0.99	6.47	±	1.74
#NEUT (10^9^/L)	0.65	±	0.17	1.31	±	0.56**↑	1.14	±	0.36*↑	1.53	±	0.62**↑	1.62	±	1.39	0.74	±	0.15	0.98	±	0.21	0.90	±	0.34
#LYMPH (10^9^/L)	4.11	±	0.88	4.17	±	0.88	5.12	±	1.53	4.13	±	0.79	3.65	±	1.65	2.75	±	0.63	4.33	±	1.01	5.08	±	1.43
#MONO (10^9^/L)	0.30	±	0.07	0.41	±	0.12	0.51	±	0.13**↑	0.46	±	0.19	0.39	±	0.19	0.26	±	0.08	0.40	±	0.11	0.37	±	0.07
#EOS (10^9^/L)	0.06	±	0.01	0.08	±	0.03	0.09	±	0.02*↑	0.12	±	0.06	0.06	±	0.03	0.07	±	0.02	0.11	±	0.02*↑^▴^↑	0.11	±	0.04*↑^▴^↑
#BASO (10^9^/L)	0.01	±	0.01	0.01	±	0.00	0.02	±	0.01**↑	0.01	±	0.01	0.01	±	0.01	0.00	±	0.01	0.01	±	0.00	0.01	±	0.00
%NEUT (%)	12.7	±	2.9	21.8	±	6.7**↑	17.2	±	6.4	24.1	±	6.8**↑	25.7	±	16.4	20.0	±	6.1	17.2	±	4.8	13.8	±	3.1
%LYMPH (%)	80.0	±	3.5	69.7	±	7.4**↓	73.7	±	6.6*↓	66.7	±	7.3 **↓	66.3	±	15.8	71.5	±	6.4	73.8	±	6.3	78.2	±	4.5
%MONO (%)	5.8	±	0.8	7.0	±	1.6	7.5	±	1.2	7.2	±	1.8	6.7	±	1.0	6.6	±	1.4	6.9	±	1.9	6.0	±	2.1
%EOS (%)	1.3	±	0.4	1.4	±	0.5	1.3	±	0.4	1.8	±	0.8	1.2	±	0.5	1.7	±	0.3	1.9	±	0.4	1.8	±	0.4
%BASO (%)	0.2	±	0.1	0.2	±	0.1	0.3	±	0.1	0.2	±	0.1	0.1	±	0.1	0.1	±	0.2	0.2	±	0.0	0.1	±	0.1
RBC (10^12^/L)	8.17	±	0.25	7.83	±	0.27*↓	7.77	±	0.37**↓	7.79	±	0.30**↓	7.79	±	0.21	7.82	±	0.21	7.90	±	0.45	7.65	±	0.15
HGB (g/L)	153	±	6	150	±	7	150	±	5	150	±	5	148	±	5	149	±	5	148	±	7	150	±	5
HCT (%)	43.9	±	1.5	43.2	±	1.6	43.0	±	1.4	42.8	±	1.5	42.4	±	1.2	42.4	±	1.1	42.8	±	2.0	42.2	±	1.3
MCV (fL)	53.7	±	1.0	55.1	±	0.9*↑	55.4	±	1.4**↑	55.0	±	1.6*↑	54.4	±	1.7	54.3	±	1.4	54.2	±	0.8	55.2	±	2.1
MCH (pg)	18.7	±	0.4	19.1	±	0.5	19.3	±	0.5**↑	19.3	±	0.5 **↑	19.1	±	0.9	19.0	±	0.6	18.8	±	0.4	19.5	±	0.8
MCHC (g/L)	348	±	3	346	±	5	349	±	4	350	±	4	350	±	6	351	±	4	346	±	4	354	±	3
RDW-CV (%)	13.1	±	0.4	13.1	±	0.8	12.6	±	0.9	12.7	±	0.7	13.4	±	0.7	13.5	±	0.7	13.2	±	0.7	13.2	±	0.5
PLT (10^9^/L)	1119	±	178	1115	±	102	1043	±	118	1073	±	72	1098	±	126	1042	±	107	1086	±	117	1076	±	79
MPV (fL)	7.2	±	0.3	7.1	±	0.2	7.2	±	0.3	7.1	±	0.3	7.3	±	0.2	7.3	±	0.3	7.3	±	0.2	7.4	±	0.2
#RETIC (10^12^/L)	0.23	±	0.03	0.27	±	0.05*↑	0.23	±	0.03^▴▴^↓	0.25	±	0.03	0.24	±	0.04	0.21	±	0.04	0.23	±	0.02	0.21	±	0.04
%RETIC (%)	2.9	±	0.4	3.5	±	0.6**↑	2.9	±	0.3^▴^↓	3.3	±	0.4*↑	3.1	±	0.4	2.7	±	0.6	2.9	±	0.3	2.7	±	0.5
PT(s)	8.9	±	0.4	8.8	±	0.3	8.6	±	0.4	8.7	±	0.3	8.7	±	0.1	8.9	±	0.1	8.9	±	0.6	8.5	±	0.5
Fbg (g/L)	1.943	±	0.137	2.612	±	0.342**↑	2.760	±	0.290**↑	3.000	±	0.194**↑^▴▴^↑	1.964	±	0.275	1.669	±	0.209	1.891	±	0.148	1.987	±	0.181
APTT (s)	16.6	±	1.2	16.7	±	1.1	16.4	±	1.0	16.3	±	1.3	17.1	±	0.7	17.2	±	0.7	17.3	±	0.8	15.8	±	2.0

Compared with the blank control group, ^*^P < 0.05, **P < 0.01. Compared with the adjuvant control group, ^▴^P < 0.05, ^▴▴^P<0.01.

**Table 2:** The hematology analysis in male SD rats treated with herpes zoster vaccine (CHO cell).

Parameter	Post-vaccination period (n = 10)												End of the recovery period (n = 5)											
	Blank control			Adjuvant control			Low-dose			High-dose			Blank control			Adjuvant control			Low-dose			High-dose		
WBC (10^9^/L)	9.28	±	1.92	8.52	±	1.75	8.44	±	1.76	9.19	±	2.28	8.67	±	2.29	8.48	±	2.07	9.85	±	2.20	7.97	±	0.47
#NEUT (10^9^/L)	1.35	±	0.60	1.67	±	0.56	1.46	±	0.42	2.57	±	0.91**↑^▴▴^↑	1.25	±	0.38	1.73	±	0.69	2.04	±	0.93	1.29	±	0.41
#LYMPH (10^9^/L)	7.27	±	1.47	6.12	±	1.77	6.29	±	1.38	5.79	±	1.54	6.86	±	1.95	5.94	±	1.55	7.00	±	1.15	6.05	±	0.34
#MONO (10^9^/L)	0.55	±	0.22	0.62	±	0.14	0.60	±	0.15	0.71	±	0.20	0.46	±	0.12	0.70	±	0.32	0.67	±	0.21	0.49	±	0.09
#EOS (10^9^/L)	0.09	±	0.03	0.10	±	0.03	0.08	±	0.04	0.10	±	0.03	0.07	±	0.03	0.10	±	0.08	0.12	±	0.05	0.13	±	0.03
#BASO (10^9^/L)	0.02	±	0.01	0.02	±	0.01	0.02	±	0.01	0.02	±	0.01	0.02	±	0.01	0.02	±	0.01	0.02	±	0.01	0.02	±	0.01
%NEUT (%)	14.2	±	4.4	20.5	±	8.2	17.2	±	3.0	27.8	±	6.8**↑	14.6	±	3.8	20.2	±	5.4	19.9	±	5.1	16.0	±	4.6
%LYMPH (%)	78.7	±	6.0	70.7	±	8.9* ↓	74.4	±	4.0	63.1	±	6.6**↓^▴^↓	79.0	±	3.5	70.4	±	8.4	71.8	±	5.1	76.0	±	4.3
%MONO (%)	6.0	±	2.3	7.4	±	1.6	7.2	±	2.0	7.8	±	1.0	5.4	±	0.6	8.1	±	2.5	6.9	±	1.5	6.2	±	1.2
%EOS (%)	1.0	±	0.3	1.2	±	0.5	1.0	±	0.4	1.1	±	0.3	0.8	±	0.2	1.1	±	0.8	1.2	±	0.3	1.6	±	0.3
%BASO (%)	0.2	±	0.1	0.2	±	0.1	0.2	±	0.1	0.2	±	0.1	0.1	±	0.1	0.2	±	0.1	0.2	±	0.1	0.2	±	0.1
RBC (10^12^/L)	8.94	±	0.45	8.49	±	0.37	8.74	±	0.49	8.69	±	0.43	8.97	±	0.55	9.01	±	0.25	8.86	±	0.65	8.92	±	0.47
HGB (g/L)	160	±	5	156	±	5	158	±	6	158	±	6	161	±	8	162	±	5	160	±	12	162	±	9
HCT (%)	46.9	±	1.5	45.7	±	1.4	46.0	±	1.7	45.3	±	1.3	47.2	±	2.4	47.3	±	1.2	46.5	±	3.1	47.0	±	2.2
MCV (fL)	52.4	±	1.3	53.8	±	2.2	52.7	±	1.4	52.2	±	1.7	52.6	±	1.4	52.5	±	1.6	52.5	±	1.3	52.7	±	0.9
MCH (pg)	17.9	±	0.6	18.4	±	0.8	18.1	±	0.5	18.2	±	0.6	18.0	±	0.7	18.0	±	0.6	18.0	±	0.6	18.1	±	0.4
MCHC (g/L)	341	±	5	342	±	6	343	±	3	348	±	5**↑^▴^↑	342	±	4	342	±	5	344	±	4	344	±	4
RDW-CV (%)	15.6	±	1.0	14.9	±	1.1	15.7	±	1.2	15.8	±	0.9	15.9	±	1.3	16.7	±	0.4	16.1	±	0.9	16.3	±	0.7
PLT (10^9^/L)	1143	±	78	1151	±	98	1185	±	141	1115	±	76	1214	±	123	1164	±	85	1103	±	95	1159	±	50
MPV (fL)	7.0	±	0.3	7.1	±	0.2	7.1	±	0.4	7.4	±	0.3*↑^▴^↑	7.3	±	0.1	7.1	±	0.2	7.3	±	0.2	7.1	±	0.2
#RETIC (10^12^/L)	0.27	±	0.03	0.28	±	0.06	0.29	±	0.03	0.25	±	0.02	0.26	±	0.04	0.28	±	0.03	0.25	±	0.03	0.24	±	0.02
%RETIC (%)	3.0	±	0.4	3.3	±	0.7	3.4	±	0.3	2.9	±	0.3	3.0	±	0.4	3.1	±	0.3	2.8	±	0.5	2.8	±	0.3
PT(s)	10.9	±	0.9	10.2	±	0.9	9.8	±	0.5	9.9	±	1.1	9.7	±	0.5	9.8	±	0.6	10.3	±	1.0	10.3	±	1.1
Fbg (g/L)	2.673	±	0.250	2.718	±	0.971	3.215	±	0.275*↑^▴^↑	4.008	±	0.248**↑^▴▴^↑	2.393	±	0.317	2.255	±	0.213	2.411	±	0.200	2.158	±	0.077
APTT (s)	19.4	±	2.1	18.3	±	2.1	17.4	±	1.4	17.9	±	1.9	18.1	±	1.4	18.5	±	1.6	19.3	±	3.0	20.2	±	1.5

Compared with the blank control group, ^*^P < 0.05, ^**^P < 0.01. Compared with the adjuvant control group, ^▴^P < 0.05, ^▴▴^P<0.01..

Aside from the differences mentioned above, no other test article-related changes in hematological parameters were observed in any group during either phase of examination.

**Figure 3:**
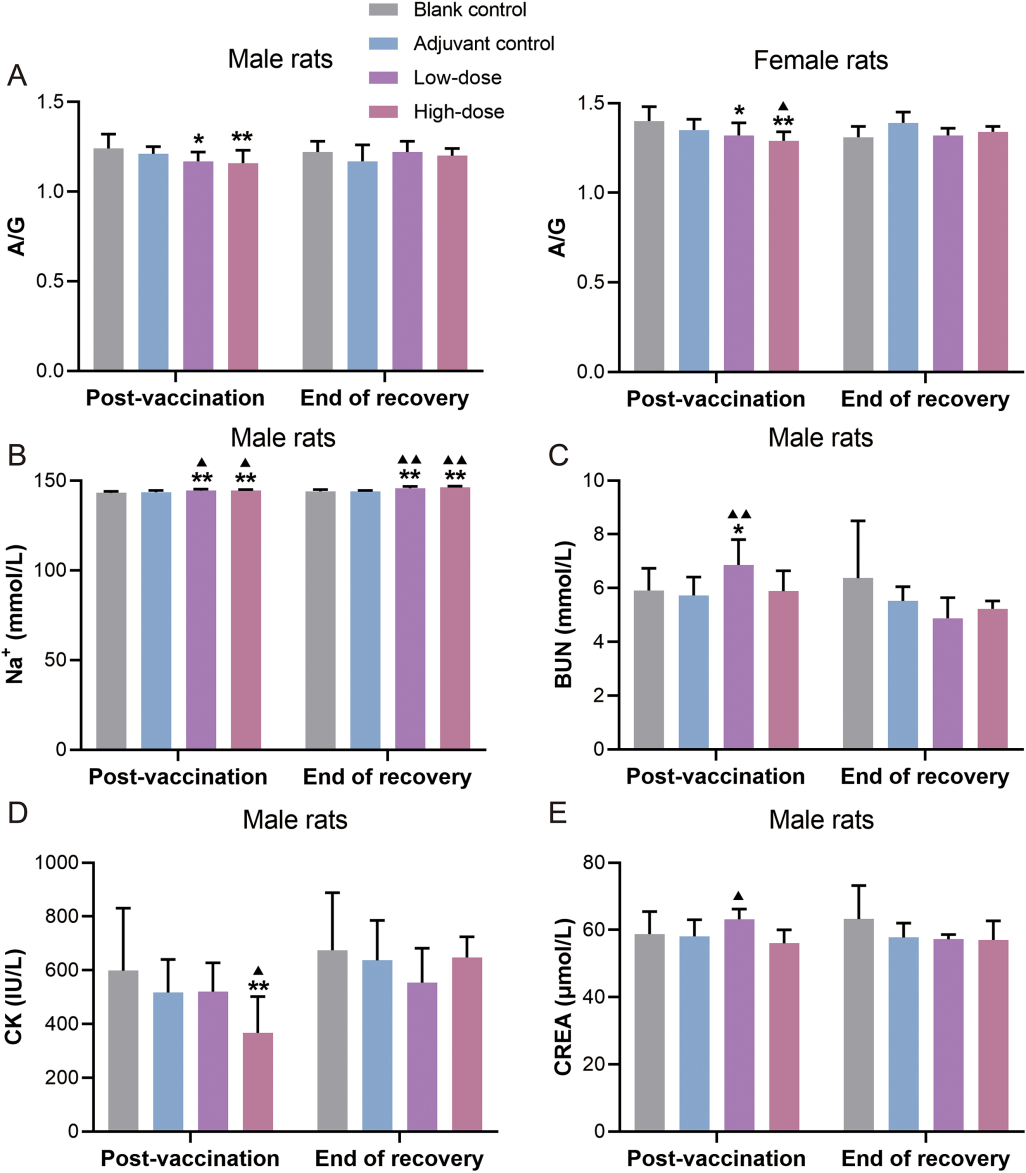
Serum biochemical analysis of recombinant zoster vaccine (CHO cell) intramuscularly injected into rats for 9 weeks of repeated administration. **(A)** A/G, albumin/globulin ratio; **(B)** Na+, sodium; **(C)** BUN, blood urea nitrogen; **(D)** CK, creatine kinase; **(E)** CREA, creatinine. Compared with the blank control group, *P < 0.05, **P < 0.01. Compared with the adjuvant control group, ^▴^P < 0.05, ^▴▴^P < 0.01. Post-vaccination period (n = 10/gender), end of the recovery period (n = 5/gender).

Compared with the blank control group, although a slight decrease in the albumin/globulin (A/G) ratio was observed in the low- and high-dose vaccine groups at the post-vaccination period, the change was considered to be devoid of toxicological significance. Variations were also noted in several biochemical parameters (decreased creatine kinase [CK↓] and increased creatinine [CREA], blood urea nitrogen [BUN], and sodium [Na^+^↑]) during both examination phases (**Figure 3**). These alterations were mild and regarded as fluctuations within the normal range of assay variation, with no apparent toxicological relevance. Overall, no test article-related adverse effects on serum biochemical parameters were observed in rats in either phase of this study.

### 3.3 Urinalysis and ophthalmic examination

Compared with the blank control group, no test article-related abnormalities were detected in the urinalysis or ophthalmological indices at any time point: before RZV vaccination, at post-vaccination, or after recovery. These data are not presented.

### 3.4 Immunotoxicity indicators

**Figure 4:**
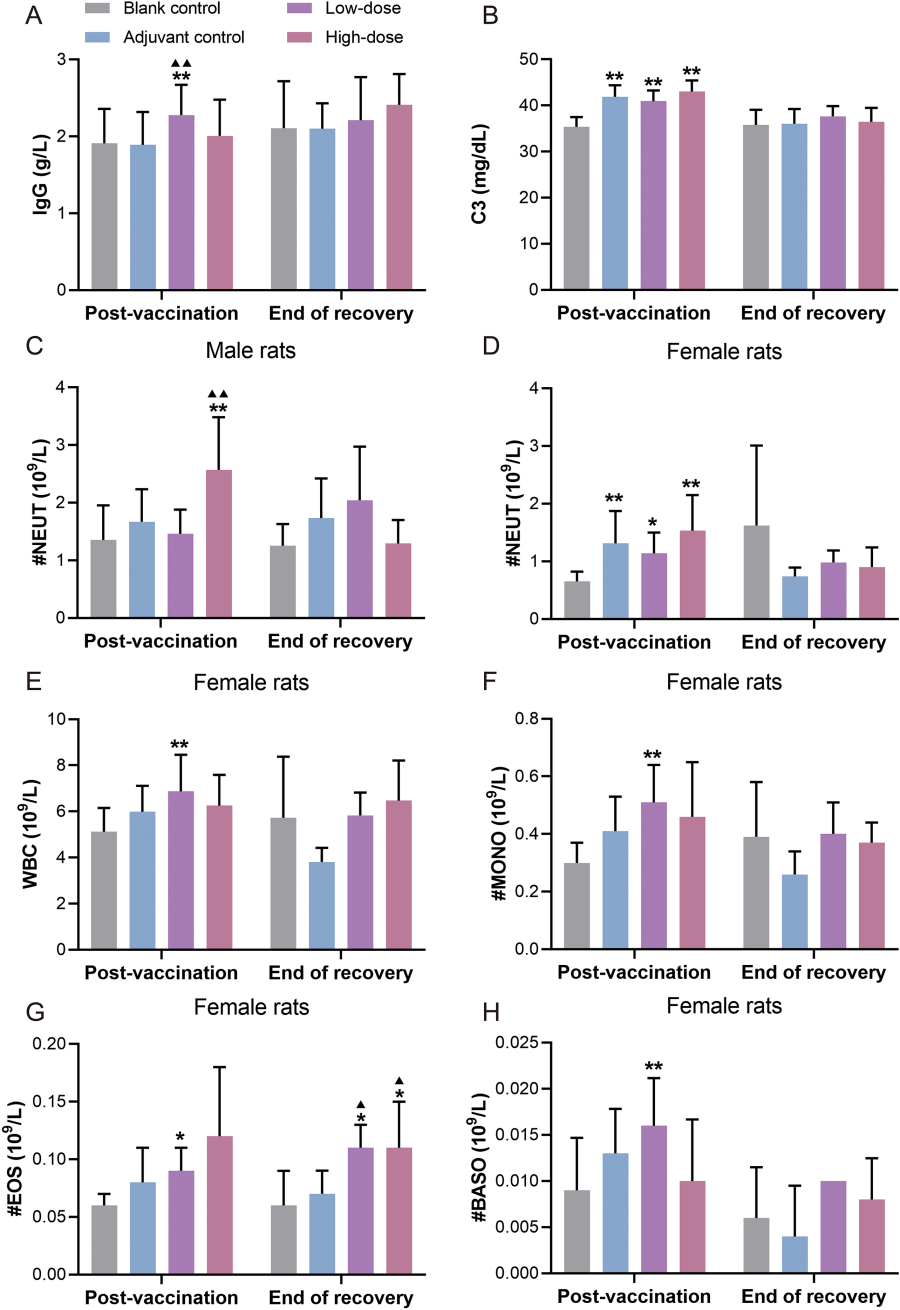
Immunotoxicity indicators of recombinant zoster vaccine (CHO cell) intramuscularly injected into rats for 9 weeks of repeated administration. **(A)** IgG; **(B)** C3; **(C–H)** white blood cell-related parameters, including neutrophils (NEUT), white blood cell count (WBC), monocytes (MONO), eosinophils (EOS), and basophils (BASO). Compared with the blank control group, ^*^P < 0.05, ^**^P  < 0.01. Compared with the adjuvant control group, ^▴^P < 0.05, ^▴▴^P  < 0.01. Post-vaccination period (n = 10/gender), end of the recovery period (n = 5/gender).

Compared with the blank control group, the low- and high-dose groups demonstrated an increasing trend in IgG levels at the post-vaccination period (**Figure 4A**). Elevations in C3 levels were observed in the adjuvant control, low-, and high-dose groups (**Figure 4B**). Neutrophil (NEUT) levels increased in high-dose female and male rats, as well as in low-dose female rats, with a similar trend noted in female rats of the adjuvant control group; these changes were no longer observed after the 4-week recovery period. Other white blood cell-related parameters (WBC, MONO, EOS, and BASO) were also slightly elevated in the low-dose female rats. At the end of recovery, eosinophil counts (EOS) remained elevated in females in the low- and high-dose groups compared with those in the blank and adjuvant controls. No other notable changes were observed in the total leukocyte count, differential leukocyte count, or cell proportion (**Figures 4C–H**).

No remarkable abnormalities were detected in the peripheral blood lymphocyte immunophenotypes, immune organ (spleen and thymus) weights, or organ-to-body weight ratios across the groups. Despite the differences reported, all other immunotoxicity indicators were comparable to the blank control at the corresponding time points. Collectively, these observations are attributable to immune activation and local inflammatory reactions after vaccination.

### 3.5 Immunogenicity

**Figure 5:**
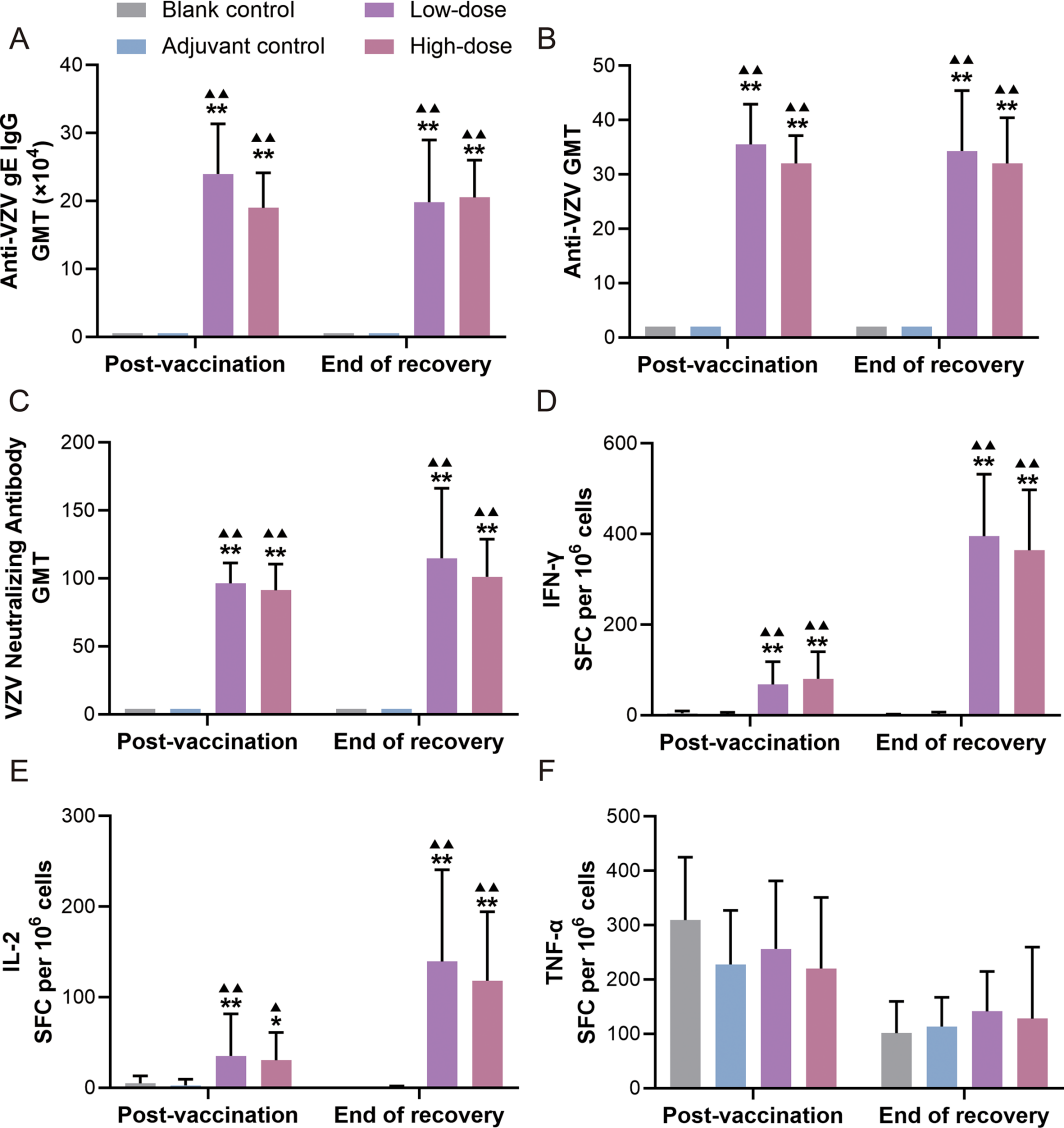
Immunogenicity results of recombinant zoster vaccine (CHO cell) intramuscularly injected into rats for 9 weeks of repeated administration. **(A)** VZV gE-specific IgG antibody, **(B)** VZV-specific antibody, **(C)** VZV neutralizing antibody, and **(D–F)** the expression of IFN-γ, IL-2, and TNF-α. Compared with the blank control group, ^*^P < 0.05, ^**^P  < 0.01. Compared with the adjuvant control group, ^▴^P < 0.05, ^▴▴^P  < 0.01. Post-vaccination period (n = 10/gender), end of the recovery period (n = 5/gender).

In terms of humoral immunity, administration of four doses of RZV to SD rats effectively enhanced serum levels of VZV gE-specific IgG antibodies, VZV-specific antibodies, and pseudovirus-neutralizing antibodies (against the prototype strain) in high- and low-dose groups. No significant differences were observed between the post-vaccination and end of recovery periods, indicating a strong and sustained humoral immune response induced by the vaccine (**Figures 5A-C**).

Regarding cellular immunity, we have to rely on indirect parameters, such as cytokine secretion by immune cells. The four-dose vaccination significantly enhanced splenic lymphocyte secretion of antigen-specific IFN-γ and IL-2 in both dose groups. Cytokine levels were higher at recovery than at post-vaccination, demonstrating durable and potent cellular immune responses (**Figures 5D, E**). TNF-α was expressed in all groups without consistent intergroup trends (**Figure 5F**). In summary, RZV effectively stimulated potent cellular and humoral immune responses in SD rats, demonstrating favorable immunogenicity.

### 3.6 Organ weights and organ coefficients

The organ weights and coefficients for each phase are presented in **Supplementary Tables 16-19**. Compared with the blank control and adjuvant control groups, the adrenal gland weight and adrenal gland coefficient in female rats of the high-dose group were significantly increased (P < 0.05) at the post-vaccination period. With the exception of the aforementioned differences, no statistically significant differences (P > 0.05) were observed in the organ weights or organ coefficients among the remaining groups of female and male rats. The adrenal findings were deemed incidental rather than test article–toxic, based on minimal severity and lack of corresponding histopathologic abnormalities.

### 3.7 Necropsy and histopathology

**Figure 6:**
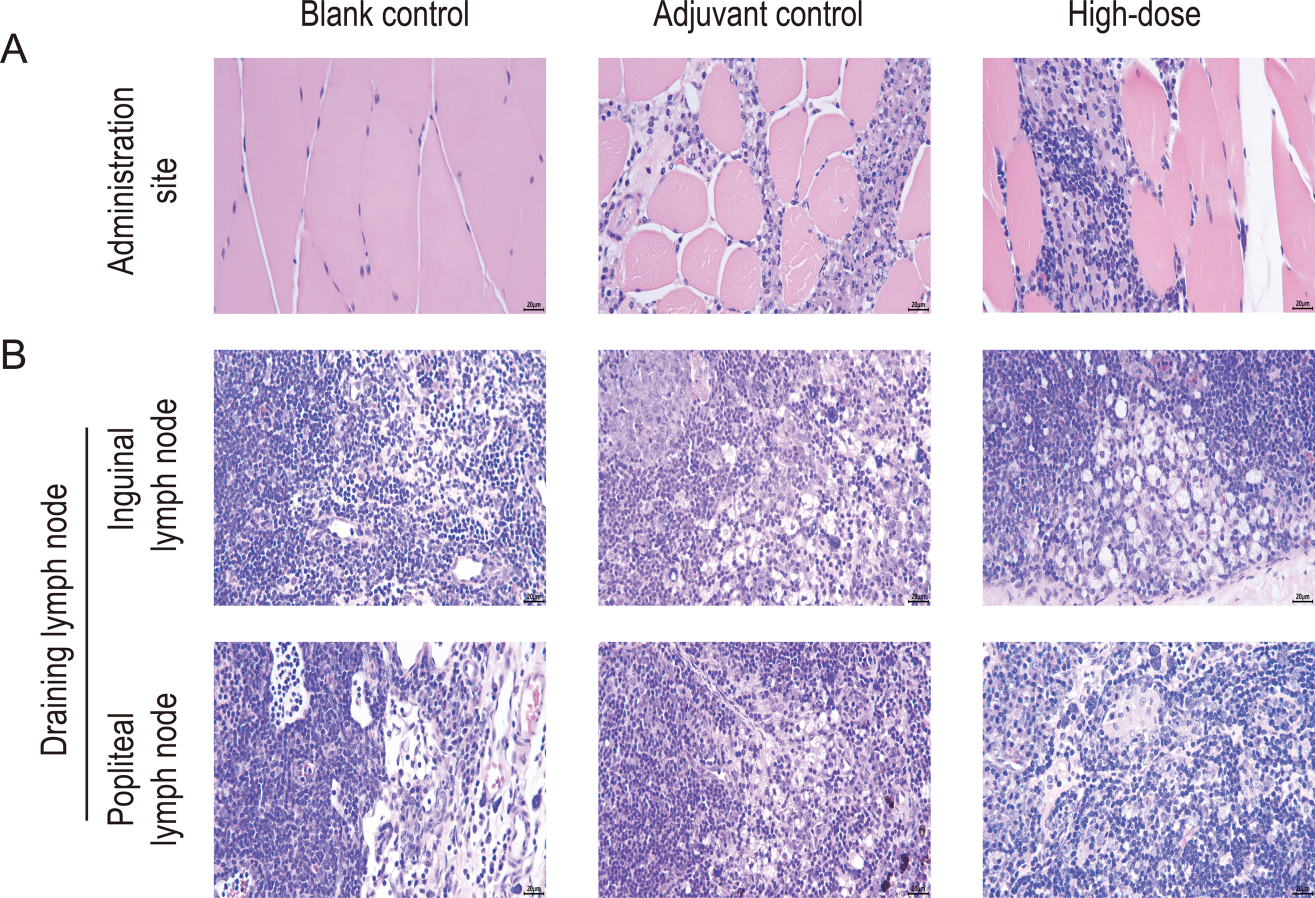
Histopathological results of rats after intramuscular injection of the recombinant zoster vaccine. **(A)** Vaccination site and **(B)** draining lymph nodes (inguinal and popliteal) (400×, scale bar = 20 μm). Post-vaccination period (n = 10/gender), end of the recovery period (n = 5/gender).

No morbidity or mortality was observed in rats throughout the study. Based on combined gross and histopathological examinations, most or all animals in adjuvant control, low-, and high-dose groups demonstrated focal subacute inflammatory reactions in the intermuscular area at the vaccination site (**Figure 6A**). Furthermore, the majority of rats exhibited mild to minimal medullary macrophage hyperplasia and mild to minimal cortical germinal center hyperplasia in the draining lymph nodes (inguinal and popliteal) (**Figure 6B**). After the 4-week recovery period, injection-site and draining lymph node changes persisted in the adjuvant control, low-dose, and high-dose groups, although they displayed a trend toward resolution. These findings were attributed to the aluminum-containing diluent and were typical local reactions to intramuscular aluminum-containing formulations. No other test article-related abnormal pathological changes were observed in the remaining tissues or organs of any of the rats during either phase of the examination.

## 4 Discussion

Population aging has been accompanied by a higher incidence of herpes zoster globally and in China, with growing costs to healthcare systems. Vaccination is the most effective preventive measure, although coverage in China remains limited. A national survey indicated insufficient uptake among adults aged ≥ 50 years, driven by hesitancy related to cost, knowledge gaps, and trust (Xia et al., 2025). For immunocompromised individuals, the recombinant zoster vaccine serves as a safe and effective option with the potential to alleviate health and socioeconomic burdens (Ishihara et al., 2024). The ongoing development and refinement of safe and effective VZV vaccines, coupled with efforts to improve vaccination coverage, could substantially reduce the incidence of HZ, prevent severe complications, establish herd immunity, and interrupt the viral transmission. These measures would thereby alleviate healthcare costs and strengthen global immunization efforts while contributing to long-term disease control.

This study conducted a comprehensive safety assessment of the “recombinant zoster vaccine (CHO cell)” in SD rats. The results demonstrated no notable adverse reactions or animal mortality during the experiment. No significant abnormalities were observed in body weight, food consumption, urinalysis, or ophthalmological parameters throughout the study period. These findings indicate that the recombinant zoster vaccine did not exhibit significant toxicological effects on the general health or clinical indicators of rats, confirming its favorable safety profile.

Simultaneously, immunogenicity results demonstrated that RZV possesses a robust capacity to induce and enhance both humoral and cellular immune responses. Rats in the low- and high-dose vaccine groups exhibited high levels of VZV gE-specific IgG antibodies and VZV-specific antibodies at the post-vaccination period, which remained elevated even after the 4-week recovery phase. Correspondingly, immunotoxicity evaluation revealed a significant increase in serum IgG levels in low- and high-dose groups at the post-vaccination period. These changes are associated with the immune response induced by vaccination. The elevated complement C3 levels observed in adjuvant control, low-, and high-dose groups were probably related to the antigen presentation response induced by the adjuvant. Alterations in leukocyte-related parameters are thought to be linked to inflammatory and immune reactions triggered by vaccine injections. Notably, mild eosinophilia persisted in female rats of the low- and high-dose groups at the end of the 4-week recovery period, which has raised concerns regarding potential safety implications. As highlighted, this persistent eosinophilia was initially attributed to immune activation induced by RZV vaccination, and relevant hematological changes have been detailed in Section 3.2 (Hematological Indicators) and immunotoxicity-related analyses in Section 3.4 (Immunotoxicity Assessment). To further address this concern, we performed histopathological examinations of relevant tissues (e.g., lungs, gastrointestinal tract) that are typically associated with eosinophil infiltration; however, no evidence of eosinophil infiltration was identified in these tissues. Based on these findings, the persistent elevation of eosinophils in female rats of the low- and high-dose groups is considered to be an effect specifically associated with vaccine-induced immune activation, rather than a pathological response involving tissue injury or infiltration. This is consistent with the overall immunogenic profile of RZV, as the vaccine is designed to elicit a robust immune response, and transient or persistent changes in immune-related cell populations (such as eosinophils) may represent a normal adaptive immune reaction to the vaccine antigen, without indicating overt toxicity.

Notably, the candidate RZV evaluated in this study incorporated a novel BC02 adjuvant system manufactured using an innovative preparation process (Qiuhong et al., 2024). Unlike traditional aluminum-based adjuvants, BC02 does not lead to detectable deposition at the intramuscular injection site (He et al., 2015; Sun et al., 2025). It can be readily mixed with the antigen extemporaneously (ready-to-use) and acts synergistically to enhance the immunogenicity. This adjuvant system allows for a reduced antigen dosage, accelerates the immune response to antigenic components, and prolongs the duration of immunity, thereby improving the vaccine efficacy in preventing herpes zoster.

Although our preclinical data demonstrate encouraging safety and immunogenicity profiles, large-scale and long-term clinical studies are required to confirm the durability of the immune response and monitor potential rare adverse events.

## 5 Conclusion

Under the conditions of this study, repeated intramuscular administration of the “recombinant herpes zoster vaccine (CHO cell)” for nine weeks produced no significant toxic effects in rats, other than changes observed in the adjuvant control group. No clear target organs of toxicity were identified, and the NOAEL was 1.0 mL per rat, equivalent to two human doses. The vaccine induced strong cellular and humoral responses, indicating the favorable immunogenicity of the vaccine.

These findings substantiate the safety profile of the “recombinant zoster vaccine (CHO cell)” and support its progression to human clinical trials. The candidate vaccine received clinical trial approval from the NMPA of China (Notification No. 2025LP01740), authorizing the evaluation of adults aged ≥ 40 years for preventing herpes zoster.

## Data Availability

The original contributions presented in the study are included in the article/**Supplementary Material**. Further inquiries can be directed to the corresponding authors.
